# Recent developments and future directions for competing stimulus assessments

**DOI:** 10.1002/jaba.70074

**Published:** 2026-07-29

**Authors:** Samantha L. Breeman, Casey Irwin Helvey, Brian D. Greer

**Affiliations:** ^1^ Rutgers Brain Health Institute Piscataway NJ USA; ^2^ Children's Specialized Hospital–Rutgers University Center for Autism Research Education, and Services (CSH–RUCARES) Somerset NJ USA; ^3^ Department of Pediatrics Rutgers Robert Wood Johnson Medical School New Brunswick NJ USA

**Keywords:** challenging behavior, competing stimuli, competing stimulus assessment, review

## Abstract

Competing stimulus assessments (CSAs) are designed to identify stimuli that reduce challenging behavior through reinforcer competition or substitution. Haddock and Hagopian (2020) found that CSAs demonstrate utility across functions and topographies of challenging behavior. Stimuli identified by CSAs often performed similarly in validation tests. Since that review, augmented CSA (A‐CSA) procedures have demonstrated efficacy in identifying greater numbers of competing stimuli compared with free‐access tests. Given these rapid advancements, another review appears warranted. The current review analyzed individual participant data to measure the prevalence, procedural characteristics, efficacy, and predictive validity of CSAs and A‐CSAs across functions and use of high‐competition stimuli in treatment packages. Approximately one third of applications used augmented procedures. Applications for socially reinforced behavior and subsequent treatment evaluations appear to be increasing. The efficacy of A‐CSAs for automatically maintained behavior suggests that augmentations for socially reinforced behavior may prove fruitful. Future directions for CSA research are discussed.

Competing stimulus assessments (CSAs) are designed to identify stimuli that compete with challenging behavior through either reinforcer competition or substitution (Piazza et al., 1998). There are several procedural elements that differentiate CSAs from preference assessments. CSAs typically include a control trial in which there is no stimulus available. Second, across both test and control trials, the relevant establishing operations and consequences are present for either socially or automatically maintained behavior. Finally, data are collected for targeted challenging behavior as well as engagement with test stimuli. It has been suggested that when reductions in challenging behavior are observed, engagement with test stimuli produces reinforcement that competes with or substitutes the functional reinforcer for challenging behavior. Stimuli that produce reduced levels of challenging behavior by at least 80% relative to no‐stimulus control trials, commonly referred to as high‐competition (HC) stimuli, are then noncontingently available during treatment (e.g., Jeglum et al., 2022).

In their review of the literature on CSAs, Haddock and Hagopian (2020) identified 15 articles reporting on 23 participants and 26 CSA applications that were published over a period spanning from 1998 to 2019. They reported that researchers most often conducted CSAs for automatically maintained behavior. This was perhaps unsurprising given CSAs evolved from pica assessments (Piazza et al., 1998). More surprisingly, Haddock and Hagopian found that high levels of stimulus engagement did not necessarily correlate with greater reductions in challenging behavior and vice versa. Despite this, the reviewed CSAs often identified at least one HC stimulus. Moreover, when validation^1^ data were available, CSA results often predicted which stimuli competed under extended evaluations.

However, CSAs do not always yield positive results (Hagopian et al., 2020). In response, Hagopian et al. (2020) evaluated a systematic manner of progressively augmenting CSA (referred to as A‐CSA) procedures. By adding prompting to encourage engagement with stimuli and then combining prompting for engagement with response blocking of challenging behavior (as necessary), Hagopian et al. demonstrated that A‐CSAs identified a greater proportion of competing stimuli than a free‐access condition for six participants. Note, the free‐access condition consisted of traditional CSA procedures (i.e., no prompting or response blocking). Further, increases in stimulus engagement and decreases in challenging behavior were largely maintained during a repeated free‐access condition following exposure to the augmented procedures. Although this was not the first example of modifying CSA procedures (e.g., Jennett et al., 2011), Hagopian et al. presented a clear demonstration of how CSAs could be made more efficacious for individuals displaying treatment‐resistant subtypes of self‐injury.

Leif et al. (2020) also described a CSA modification that involved prompting and differential reinforcement in lieu of response blocking to increase stimulus engagement within A‐CSAs. Similarly, Schmidt et al. (2021) evaluated prompting and response blocking augmentations in test trials for both leisure stimuli and tasks.^2^ Although these examples represent some of the novel augmentations in the literature to date, most researchers evaluating the A‐CSA implemented the procedures outlined by Hagopian et al. (2020) and replicated by Frank‐Crawford et al. (2023). It is also important to note that the terminology to describe competing stimuli has since been revised to account for procedures that involve response blocking. Specifically, researchers recommend using the term HC stimuli only when discussing stimuli producing an 80% or greater reduction in challenging behavior that were identified under free‐access or prompting conditions because reductions in challenging behavior when response blocking or disruption is used cannot be isolated from the effects of the competing stimulus.

Laureano et al. (2023) used a retrospective consecutive controlled case series (CCCS) to examine CSA efficacy for every clinical case for which a CSA was conducted in an inpatient setting. Their data sets included traditional CSAs and the free‐access condition from A‐CSAs. They found CSAs identified at least one HC stimulus in approximately 47.20% of applications, which was well below the 92.30% observed in Haddock and Hagopian (2020). However, there are a few differences in their procedures and respective data sets worth noting. Inclusion criteria varied across studies in that Laureano et al. reviewed all clinical data sets that met the inclusion criteria regardless of responding, whereas Haddock and Hagopian reviewed only published data sets, which may favor inclusion of cases with positive outcomes.

Moreover, Laureano et al. (2023) analyzed their data with respect to behavioral function and identified two pertinent differences in the composition of functions across applications relative to those described in Haddock and Hagopian (2020). First, Laureano et al. included substantially more applications involving Subtype 2 and 3 automatically maintained self‐injurious behavior (SIB; 52.8%) relative to Haddock and Hagopian (3.8%). Laureano et al. found that the CSA identified at least one HC stimulus for Subtype 1 automatically maintained SIB in 66.7% of applications, which was comparable to the percentage identified for socially maintained behavior (60.0%). Conversely, at least one HC stimulus was identified for Subtype 2 and 3 automatically maintained SIB in only 45.0% and 25.0% of applications, respectively. These subtypes have historically been associated with greater difficulty identifying HC stimuli relative to Subtype 1 using the CSA (e.g., Frank‐Crawford et al., 2023; Hagopian et al., 2015; Laureano et al., 2023), but A‐CSA procedures can improve outcomes for Subtypes 2 and 3 (Frank‐Crawford et al., 2023). Second, the proportion of applications involving challenging behavior maintained by mixed reinforcement (i.e., automatic and social) was substantially higher in Laureano et al. (41.5%) than in Haddock and Hagopian (11.0%). The CSA identified at least one HC stimulus in only 36.4% of applications in the mixed reinforcement group compared to 53.8% and 60.0% of applications in the automatic and social reinforcement groups, respectively. Laureano et al. suggested that identifying HC stimuli may be more difficult for individuals with challenging behavior maintained by mixed reinforcement because the competing stimulus must compete with two distinct sources of reinforcement. Thus, the greater proportion of Subtype 2 and 3 automatically maintained SIB and mixed reinforcement functions included in Laureano et al. may have contributed to the lower overall effectiveness of the CSA relative to Haddock and Hagopian. These findings suggest that analyzing responding within behavioral function groups in the published literature may be warranted.

Examining CSA procedures, especially when applied to socially reinforced behavior, remains an important area given the differences in assessment and their use in treatment relative to CSAs for automatically maintained challenging behavior. Specifically, CSAs for socially reinforced behavior require implementers to arrange the relevant establishing operation(s) for challenging behavior and deliver the maintaining reinforcer(s) contingent on challenging behavior to examine the extent to which the test stimuli compete with the maintaining reinforcer (e.g., Fisher et al., 2004). However, no previous review on CSAs has explicitly examined the reinforcement contingencies arranged for challenging behavior within the assessment, such as the schedule of reinforcement for challenging and alternative behavior (if applicable). In addition, some validations for socially reinforced behavior differ in that implementers provide access to the HC stimuli only during delays to the functional reinforcer (e.g., Miller et al., 2022) rather than noncontingently throughout the session, raising questions about the durability of HC stimuli during schedule thinning. Further, A‐CSAs have demonstrated efficacy with automatically maintained behavior, but it is unclear whether augmented procedures have been applied to socially reinforced behavior and, if so, whether these applications demonstrate similar efficacy.

Although the Haddock and Hagopian (2020) findings indicate that CSAs generally have strong predictive validity, no reviews have evaluated the predictive validity of A‐CSA results. Additionally, collecting data on the validation of stimuli that are not identified as HC stimuli may help researchers determine whether CSAs yield false‐negative results. Moreover, the consistency across repeated administrations of CSA and A‐CSA findings has not been documented in previous reviews. These measures are necessary for determining the full breadth of CSA utility and pinpointing directions for future researchers.

Given the recent and rapid advancements in the literature regarding CSA and A‐CSA procedures; the ambiguity of how such procedures are used for socially reinforced challenging behavior; and existing gaps regarding durability, reliability, and validity of these procedures; a systematic review of publications during this time appears warranted. The current review sought to summarize data on the (a) efficacy of CSAs and A‐CSAs, (b) relative use and efficacy of CSA applications across behavioral functions, (c) predictive validity of CSAs and A‐CSAs across behavioral functions, (d) relative frequency of repeated testing procedures to evaluate consistency of assessment results, and (e) use of HC stimuli within treatment packages.

## METHOD

### Article search

The Preferred Reporting Items for Systematic Reviews and Meta‐Analyses Individual Participant Data (PRISMA‐IPD; Stewart et al., 2015) checklist was used to guide and document the search process. Use of this methodology both replicated the procedures of Haddock and Hagopian (2020) and allowed for systematic analysis of individual participant data given the potential variability across published procedures. To the extent possible, we replicated the search procedures from Haddock and Hagopian, including the electronic databases, hosts, and search terms. However, only five of the seven databases used by Haddock and Hagopian were accessible through the first author's university library, and the authors did not conduct a subsequent Google Scholar search. In addition, one search term (i.e., “competing task”) was added to capture studies employing CSAs to identify competing tasks. Search terms, including synonyms and grammatical variations, were (1) competing stimulus OR competing items OR competing task assessment AND behavior or (2) preference assessment AND problem behavior. See Supporting Information for detailed search information. The search was restricted to articles published between 2019 and 2024. Microsoft Excel was used to compile search results and identify and remove duplicates.

### Screening of search results

The screening process included two steps: title and abstract screening followed by full‐text screening. Inclusion criteria for title and abstract screening were the same as Haddock and Hagopian (2020), including that the title or abstract indicated (a) peer‐reviewed experiment with systematic manipulation of an independent variable, (b) participants were human subjects, and (c) challenging behavior was the primary dependent variable. Note that we included case studies or reports as experiments if there was some indication in the title or abstract that there was a systematic assessment and treatment evaluation. Studies were excluded following title and abstract screening if (a) there was no experiment (e.g., book chapter, review), (b) subjects were nonhuman, or (c) challenging behavior was not the primary dependent variable.

Criteria for full‐text review were similar to Haddock and Hagopian (2020). Articles were included if at least one participant's pretreatment assessment met the definition of a CSA based on the following criteria: (a) no‐stimulus or no‐task control trial(s); (b) a series of isolated test trials during which a single stimulus was available, or a single task was presented (i.e., one stimulus or task at a time); (c) a measure of challenging behavior during all stimulus or task test and control trials; and (d) a measure of stimulus engagement, stimulus contact, or task engagement or completion during all test trials. We did not use the inclusion criteria from Haddock and Hagopian for separate and reliable reporting of challenging behavior and stimulus engagement or contact because it would have resulted in the exclusion of several relevant articles, including the pioneering article describing the application of the A‐CSAs to identify competing tasks (Schmidt et al., 2021). Articles were excluded following full‐text review if (a) a pretreatment stimulus assessment that met the definition of CSA was not conducted with at least one participant, (b) at least one CSA that met criteria was conducted but there was no graph or data table of the results, or (c) the full text was unavailable through institutional access.

### Individual participant data inclusion

Criteria a–d listed above (i.e., inclusion of control and isolated test trials and measures of challenging behavior and either stimulus engagement or contact) were applied to determine inclusion at the individual level. We included CSA validations for coding that met the same criteria as Haddock and Hagopian (2020).

### Individual participant data coding

#### Participant demographics

For each included participant in an included study, their reported sex at birth, age, race or ethnicity, diagnosis or diagnoses, topography or topographies of challenging behavior, and identified behavioral function (i.e., automatic, social, or mixed) were recorded. For social and mixed functions, we also recorded the specific classification (i.e., tangible, attention, escape, multiply maintained, or other).

#### Number of CSA applications and validations

Similar to Haddock and Hagopian (2020), distinct CSA and validation applications for a given participant were defined by the implementation of different independent or dependent variables. For example, participants who experienced CSAs targeting different topographies of challenging behavior (e.g., self‐injury and pica) or testing different stimuli were coded as two separate applications. When available, data were collected on whether these multiple applications tested the same or different stimuli. Whether an evaluation of consistency (i.e., conducting CSA trials at a later date using the same stimuli as the original series) was included was coded for all applications.

As in Haddock and Hagopian (2020), validation procedures were defined as extended analyses (i.e., for longer durations or across multiple observations) of test stimuli in the CSA or A‐CSA following completion of the assessment. These include evaluations of test stimuli or stimulus sets as stand‐alone treatments and treatment packages including test stimuli. Validations also included evaluations of stimuli not identified as HC stimuli during the assessment. Validation procedures were coded for (a) the number of stimuli validated, (b) whether schedule thinning or terminal probes were conducted, (c) the components of treatment packages, (d) the presence of other preferred stimuli within the validation context, and (e) the availability of maintenance or follow‐up treatment data. Further, the use of stimulus sets, or more than one identified HC stimuli per validation session, was recorded. We included validations wherein experimenters rotated the available HC stimuli during the session in this definition when the individual effect of each HC stimulus could not be determined.

#### Stimulus characteristics

The type of stimulus or task assessed (e.g., leisure, food, attention) was recorded. Only one article evaluated the use of a CSA for tasks in which only leisure tasks were assessed (Schmidt et al., 2021). Although Haddock and Hagopian (2020) reported on outcomes of stimuli hypothesized to produce “matched” and “unmatched” stimulation to putative consequences of challenging behavior, no comparisons between such groups were completed in the current review due to limited reporting on this in the studies reviewed.

#### CSA procedures

For each CSA application, whether the assessment was standard or augmented was recorded. *Standard* procedures were defined as those in which participants were provided free access to each test stimulus individually, without prompting or response blocking. *Augmented* procedures were defined as those in which participants experienced a free‐access phase (as described above for standard CSA) along with one or more additional conditions that involved prompted stimulus engagement or response blocking with prompted stimulus engagement. If the procedures were augmented, then the type of augmentation was also recorded, including prompted engagement, response blocking with prompted engagement, or other.

Additional procedures recorded for each CSA application included the (a) basis of stimulus selection (e.g., caregiver report, preference assessment), (b) trial duration, (c) number of series, (d) number and type of stimuli (e.g., food, leisure, attention) or tasks assessed, (e) presence of additional stimuli (e.g., noncontingent access to preferred items), (f) schedule and type (i.e., reinforcement, punishment) of consequences arranged for challenging behavior, (g) schedule and type of consequences arranged for other behavior (e.g., praise for stimulus engagement), and (h) definition of a HC stimulus (e.g., percentage of engagement, percentage of reduction in challenging behavior). Within this review, we classified stimuli according to the procedures used to identify them. Stimuli that met the associated study's criteria for identifying competing stimuli under conditions involving free access or prompted engagement were coded as *HC stimuli*, whereas stimuli that met those criteria under conditions involving response blocking combined with prompted engagement were coded as *HC stimuli with disruption*. Data on measures of engagement versus contact, as defined and specified by the authors of each study, were also collected. Generally, definitions of stimulus engagement required the participant to actively manipulate the stimulus in a manner not consistent with challenging behavior and definitions of contact required the participant to maintain physical contact with the stimulus.

### Data analysis

#### Data extraction

Data from all relevant CSA application and validation figures were extracted using WebPlotDigitizer (accessed at https://apps.automeris.io/wpd4) and validated in the same manner as Haddock and Hagopian (2020), with one exception. When applicable, validation data from treatment phases that arranged identical contingencies with and without HC stimuli were also extracted (see Predictive validity below).

#### Challenging behavior reductions

Using the extracted data, the percentage of reduction in challenging behavior was calculated for both the CSA application and validation using the same procedures as Haddock and Hagopian (2020). Similarly, the percentage of reduction in challenging behavior was compared with the percentage of engagement or contact in the CSA per test stimulus. For A‐CSAs, we compared the percentage of reduction in challenging behavior and percentage of engagement or contact across the free‐access condition and the augmented condition with the greatest observed reduction in challenging behavior. For each CSA application, we also identified the percentage of HC stimuli across functions of challenging behavior. For A‐CSAs, we compared the percentage of identified HC stimuli across the free‐access and augmented procedures.

#### Predictive validity

The procedures for calculating predictive validity were identical to Haddock and Hagopian (2020). Specifically, we subtracted the percentage of reduction in challenging behavior obtained during the relevant validation condition from the percentage of reduction obtained during the CSA or A‐CSA. Correspondence was defined as a difference of 10 percentage points or fewer, with three additional considerations. First, we excluded cases wherein the measurement of challenging behavior differed across the CSA application and validation. Second, given the inclusion of A‐CSAs, we included relevant treatment conditions with identical contingencies as one of the augmented phases of the A‐CSA. Third, in cases where the effects of HC stimuli could not be directly compared with baseline conditions (e.g., HC stimuli were only evaluated within the context of a larger treatment package), data from identical conditions with and without the HC stimuli were compared instead. Despite these modifications, only 20 of 32 validations could be analyzed.

### Interrater agreement

The second author screened all search results, and the first author screened 33.33% to assess interrater agreement. An agreement was scored if both raters included or excluded an article. Interrater agreement during the abstract screening was 78.13%. For all disagreements, both authors met and used a consensus method for determining the final inclusion or exclusion of the article. This consisted of retrieving the article, locating the source of the disagreement, reviewing the screening requirements, and discussing whether the information provided met those requirements until a consensus was reached. Both raters took the same approach to determining the eligibility of participant data (see criteria above) for 33.33% of the articles included. The agreement was 100%. Finally, the first author and an independent rater obtained agreement on the coded variables for 33.33% of included CSA applications and validations. An agreement was scored if both raters applied the same codes to individual participant data. Agreement at this stage was 96.17%. For all disagreements, both raters met, reviewed the relevant articles together, and used a consensus method (as described above) for determining the final codes applied.

## RESULTS

### Article search and screening

**FIGURE 1:**
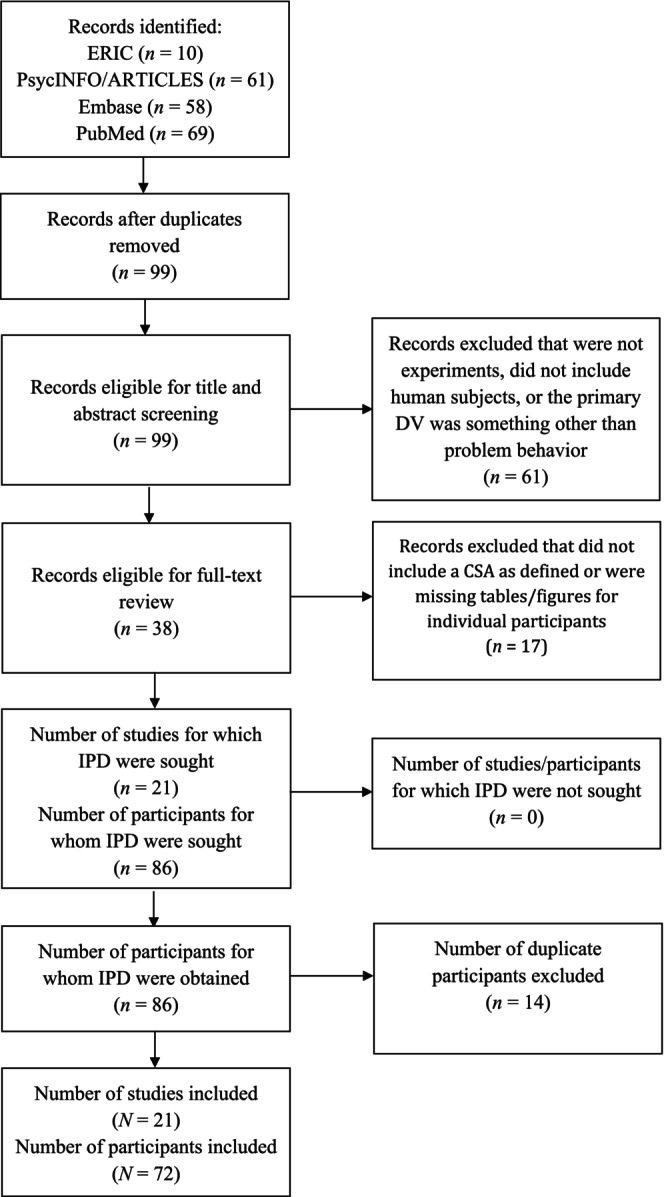
*PRISMA‐IPD* flow diagram.

Figure 1 presents the PRISMA‐IPD flow diagram summarizing the article search. Our initial search yielded 198 articles, and 99 were removed as duplicates. Thus, 99 articles were eligible for screening. Of these, 61 articles did not meet one or more of our inclusion criteria listed above and were therefore removed. The remaining 38 articles were eligible for full‐text review. Seventeen were removed that did not include a CSA (defined above in Screening of search results) or were missing data displays (e.g., tables) for individual participants. Individual participant data were sought for 21 studies. Across these, data were sought for 86 individual participants. Fourteen duplicate participant data sets were excluded for appearing in multiple publications (i.e., these data sets were retained from the original publication and excluded from the subsequent publication to avoid including the same data in our analyses twice). In total, data were obtained for 72 participants and 87 applications (defined above in Number of CSA applications and validations) for coding in the analysis.

### Individual participant data

#### Participant demographics

Table 1 displays age, sex at birth, race or ethnicity, and diagnosis for the 72 participants included across studies. The mean age of participants was 12.03 years (*Mdn* = 11.50, range: 2–27 years). Males (57, 79.17%) under the age of 18 (62, 86.11%) comprised the majority of participants. Of the participants for whom race or ethnicity was reported (*n* = 34), 22 were White (64.71%); nine were Black, African American, or Ethiopian American (26.47%); and two were Asian (5.88%). Nine participants were not Hispanic or Latino (26.47%), one participant was Hispanic or Latino (2.94%), and one participant was Brazilian (2.94%). All participants were reportedly diagnosed with at least one neurodevelopmental disorder (e.g., autism spectrum disorder, Rett syndrome) or genetic disorder (e.g., Cornelia de Lange syndrome, Lennox–Gastaut syndrome, or Kleefstra syndrome). Moreover, 54 participants (75%) had multiple diagnoses.

**TABLE 1:** Participant demographics.

Citation	Participant	Age	Sex	Race or ethnicity	Diagnosis	Challenging behavior	Function
Dowdy et al. (2020)	Shane	16	Male	Unreported	ADHD, ASD, DiGeorge syndrome, ID, Marfan's syndrome	PD	Auto
Falligant, Carver, et al. (2021)	Nick	12	Male	Unreported	ASD	DR	Ambiguous/Attn
Falligant, Hardesty, et al. (2021)	Paolo	5	Male	Unreported	Cortical visual impairment, ID, Moebius syndrome, Pierre Robin sequence,	Tracheostomy tube manipulation	Auto
Frank‐Crawford et al. (2023)	P1	12	Male	Unreported	ASD, OCD, SPD	SIB	Auto
	P2	17	Male	White, Not Hispanic	ASD, DICCD, encephalopathy SMD with SIB	SIB	Auto
	P3	10	Male	Unreported	ASD, DICCD, SMD with SIB	SIB	Auto
	P4	18	Male	Unreported	AD (NOS), ASD, DICCD, SMD with SIB	SIB	Auto
	P5	14	Male	Black	ADHD, ASD, DICCD, SMD with SIB	SIB	Auto
	P6	11	Female	Unreported	ASD, DICCD, SMD with SIB	SIB	Auto
	P7	11	Male	White, Not Hispanic	ASD, DICCD, SMD with SIB, Joubert syndrome	SIB	Auto
	P8	13	Male	White, Not Hispanic	ASD, DICCD, SMD with SIB	SIB	Auto
	P9	17	Male	White, Not Hispanic	ASD, DICCD, SMBD with SIB	SIB	Auto
	P10	10	Male	Black, Not Hispanic	AD (NOS), ADHD, ASD, DICCD, SMD with SIB, OCD	SIB	Auto
	P11	13	Female	Asian, Not Hispanic	ASD, DICCD, SMD with SIB	SIB	Auto
	P12	14	Male	White, Not Hispanic	ASD	SIB	Auto
	P13	9	Male	Unreported	ADHD, ASD, DICCD, DMDD, SMD with SIB	SIB	Auto
	P14	10	Male	White, Not Hispanic	ASD	SIB	Auto
	P15	19	Male	Unreported	ASD, SMD with SIB	SIB	Auto
	P16	10	Female	White, Not Hispanic	ASD, DICCD, SMD with SIB, Sotoc syndrome	SIB	Auto
Frank‐Crawford et al. (2024)	Winston	6	Male	Black	ASD, DBD, ID, Impulse control and conduct disorder, SMD with SIB	Elope	Tang
Hagopian et al. (2020)	1	13	Male	Unreported	ASD, OCD, SMD with SIB	SIB, SR	Auto
	2	11	Female	Unreported	ASD, ID, SMD with SIB, DBD (NOS)	SIB	Auto
	3	19	Female	Unreported	ASD, Cornelia de Lange syndrome, ID, SMD with SIB	Sty	Auto
	4	10	Male	Unreported	AD (NOS), ADHD, ID	SIB, SR	Auto
	5	5	Male	Unreported	ASD, ID	Sty	Auto
	6	21	Male	Unreported	AD (NOS), ASD, ID, MD (NOS), SMD with SIB ID,	Sty	Auto
Imler & Weyman (2024)	Tucker	12	Male	White, Brazilian	ASD	PD	Auto
	Tinley	6	Female	White	ASD	Dis Voc	Auto
	Walter	10	Male	White	ASD	PD	Auto
Jeglum et al. (2022)	Noelle	27	Female	Unreported	ASD, DBD, GERD, ID, SMD with SIB	SIB	Auto
Laureano et al. (2023)	1	10	Male	Hispanic or Latino	ADHD, ASD, SMD with SIB	SIB	Auto
	2	12	Male	White	ASD, DICCD, SMD with SIB	SIB, AGG, Dis, DR	Auto, Attn
	3	12	Male	Black or African American	ASD, Cerebral Palsy, DICCD	DR	Attn
	9	9	Male	Unreported	ADHD, ASD, DICCD, DMDD, SMD with SIB	SIB, SR, Pica	Auto, Attn
	10	18	Male	White	Monoallelic mutation of PACS1 Gene, seizure disorder, SMD with SIB, unspecified mood and anxiety disorder	AGG, Dis, Tantrum, Scream	Attn, Esc
	11	14	Male	White	ASD, OCD, SMD with SIB	SIB, AGG, Dis	Auto, Attn, Tang, Esc
	12	24	Female	White	Allergic conjunctivitis, allergic rhinitis, ASD, bipolar disorder, dyssomnia, eczema, galactorrhea, hypothyroidism, SMD with SIB	Scripting	Auto
	13	13	Male	White	ASD, chronic ear Infections and reflux, GERD, MD	SIB	Auto
	15	10	Female	White	ADHD, DMDD, SMD with SIB, suspected Pitt‐Hopkins syndrome	SIB	Attn
	17	15	Male	White	ASD, SMD with SIB	SIB, AGG, Dis	Auto, Attn
	19	20	Male	Unreported	Cornelia de Lange Syndrome	SIB	Attn
	20	15	Male	Unreported	ASD, catatonia, history of seizures, major depressive disorder, SMD with SIB	SIB	Auto
	21	11	Male	Unreported	AD, arthrogryposis, ASD, depression, encephalopathy, OCD, Schaaf–Yang syndrome, SMD with SIB	Rumination	Auto
	24	12	Male	Unreported	abnormal platelet function, arteriovenous fistula of liver, atrial septal defect, ASD, autonomic dysfunction, congenital arteriovenous malformation, hyperammonemia, hypernatremia, hypotonia, persistent left superior vena cava, partial epilepsy with impairment of consciousness, patent foramen ovale, portosystemic venous shunt, pulmonary hypertension, SMD with SIB	SIB	Attn
	25	8	Male	Asian	ASD, DICCD, seizure disorder, SMD with SIB,	SIB, AGG, Dis	Auto, Attn, Tang, Esc
	26	18	Male	White	ASD, DICCD, generalized seizure disorder, traumatic brain injury	Perseveration	Auto
	30	8	Male	Unreported	ADHD, ASD, DMDD, skin‐picking disorder, SMD with SIB,	SIB, AGG, Dis	Auto, Tang, Esc
	31	10	Male	Black or African American	AD, ADHD, ASD, DICCD, OCD, SMD with SIB	SIB, SR	Auto, Tang
	32	10	Male	White	ASD, down syndrome, mood disorder, SMD	SIB, AGG, Dis	Auto, Attn
	33	11	Male	Black or African American	ASD, SMD with SIB	Spitting	Auto
	34	13	Male	Unreported	AD, ASD	Pica, Dis, DB	Auto, Attn, Esc
Leif et al. (2020)	Julia	17	Female	Unreported	Rett syndrome	Mouthing	Auto
	Doug	10	Male	Unreported	ASD	Sty, Flop	Auto
	Tammy	11	Female	Unreported	ASD	Sty, Mouthing	Auto
	Mark	5	Male	Unreported	ASD	Mouthing	Auto
Miller et al. (2022)	Brian	5	Male	Unreported	ASD	SIB, AGG, PD	Esc, Tang
	Mark	3	Male	Unreported	ASD	SIB, AGG, PD	Esc, Tang
	Wayne	8	Male	Unreported	ASD	PD	Esc, Tang, Attn
	Keith	8	Male	Unreported	ADHD, ASD	AGG, PD	Tang
Newcomb et al. (2019)	Ted	13	Male	Unreported	ASD	AGG	Attn
Rosenzweig et al. (2024)	Brian	2	Male	Unreported	ASD	Sty	Auto
	Daniel	13	Male	Unreported	ASD	Sty	Auto
	Amy	2	Female	Unreported	ASD	Sty	Auto
	Seth	16	Male	Unreported	ASD	Sty	Auto
Ruckle et al. (2023)	Patrick	11	Male	Unreported	ASD, ID, pica	Pica	Auto
Schmidt et al. (2021)	Jeb	21	Male	Unreported	ASD	Sty	Auto
Shawler et al. (2023)	Temi	11	Male	African American	ADHD, ASD, DBD, ID, mixed receptive‐expressive language disorder	Dis, PD	Auto
Shawler et al. (2024)	Farah	3	Female	White	ASD, developmental delay, pica, restless leg syndrome, SPD,	SIB	Esc to Attn, Esc to Attn and Tang, Attn and Tang
Taylor (2020)	Herbert	4	Male	White	ASD, food selectivity, food stealing, iron deficiency, pica	Pica, Mouthing	Auto
Thomas et al. (2022)	Linda	16	Female	Black	ASD, DBD, ID, Kleefstra syndrome	AGG, SIB, Dis, PD	Auto
Thomas et al. (2023)	Maude	13	Female	White	ASD, ID	Pica	Auto
Thomas (2024)	P1	20	Male	Ethiopian American	ASD, ID, Lennox–Gastaut syndrome, SYNGAP1‐RD	AGG, SIB, PD	Auto, Tang

*Note*: AD = anxiety disorder; ADHD = attention‐deficit/hyperactivity disorder; AGG = aggression; ASD = autism spectrum disorder; Attn = attention; Auto = automatic reinforcement; DB = dangerous behavior; DBD = disruptive behavior disorder; Dis = disruption; DICCD = disruptive, impulse‐control, and conduct disorders; Dis Voc = disruptive vocalizations; DMDD = disruptive mood dysregulation disorder; DR = disrobing; Esc = escape; GERD = gastroesophageal reflux; ID = intellectual disorder; MD = mood disorder; NOS = not otherwise specified; OCD = obsessive compulsive disorder; PD = property destruction; SIB = self‐injurious behavior; SMD = stereotypic movement disorder; SPD = sensory processing disorder; SR = self‐restraint; Sty = stereotypy; Tang = tangible.

Table 1 also displays targeted challenging behavior and function for all 72 participants. Researchers used CSAs for a variety of challenging behavior, including but not limited to self‐injury, stereotypy, pica, elopement, and disrobing. In looking at behavioral function, 49 applications (68.06%) targeted automatically maintained behavior only, 13 applications (18.06%) targeted socially reinforced behavior only, and 10 applications (13.89%) targeted behavior that was both automatically maintained and socially reinforced (hereafter referred to as *mixed*). Of the 23 social and mixed function applications, 18 (78.26%) targeted an attention function, 12 (52.17%) targeted a tangible function, and 10 (43.48%) targeted an escape function. For the one participant (Nick) listed from Falligant, Carver, et al. (2021), the function was coded as attention, as the authors reported that the assessment outcome was ambiguous but that the target behavior was more likely to occur in contexts in which motivating operations for attention were present. For synthesized functions, we individually coded each function of the combined contingency. For example, if the synthesized contingency included escape to attention, then we coded one escape function and one attention function. For Shawler et al. (2024), there were multiple synthesized contingencies indicated for their participant, Farah, including escape to attention, escape to attention and tangible, and tangible and attention. In this case, we coded each time an individual function was included in a synthesized contingency. This resulted in three instances of attention, two instances of tangible, and two instances of escape. Researchers determined that nine participants' challenging behavior, targeted across 10 applications, was maintained by multiple social functions.

#### Number of CSA applications and validations

Table 2 provides the number of CSA applications and validations across participants. Thirteen participants experienced multiple CSA applications, resulting in 87 total applications. The specific stimuli or tasks tested across applications were available for seven of these participants; most (4, 57.14%) experienced CSA procedures with all new stimuli across applications. One (14.29%) experienced the procedures with one overlapping stimulus. The remaining participants (2, 28.57%) experienced the procedures with all the same stimuli but with other procedural refinements (e.g., body position of the participant and person implementing procedures). The percentage of stimuli identified as HC stimuli increased across applications for four (30.77%) participants, decreased for four (30.77%), stayed the same for two (15.38%), and remained at zero for three (23.08%).

**TABLE 2:** Number of CSA applications and validations included.

Citation	Participant	Distinct applications	Distinct validations
Dowdy et al. (2020)	Shane	1	1
Falligant, Carver, et al. (2021)	Nick	1	1 (SS)
Falligant, Hardesty, et al. (2021)	Paolo	2	2 (SS)
Frank‐Crawford et al. (2023)	P1–P3, P5, P6, P8–P13, P15, P16	1	
	P4, P7, P14	2	
Frank‐Crawford et al. (2024)	Winston	1	2
Hagopian et al. (2020)	1–6	1	
Imler & Weyman (2024)	Tucker, Tinley, Walter	1	2
Jeglum et al. (2022)	Noelle	2	1 (SS)
Laureano et al. (2023)	1, 3, 10–12, 15, 17, 19–21, 24–26, 32, 34	1	
	9, 13, 30, 33	2	
	2, 31	3	
Leif et al. (2020)	Julia	1	1
	Doug	1	2
	Tammy	1	1
	Mark	1	
Miller et al. (2022)	Brian	1	1
	Wayne	1*	1
	Mark, Keith	1	
Newcomb et al. (2019)	Ted	1	1 (SS)
Rosenzweig et al. (2024)	Brian, Daniel, Amy, Seth	1	1 (SS)
Ruckle et al. (2023)	Patrick	1	1 (SS)
Schmidt et al. (2021)	Jeb	2	2 (SS)
Shawler et al. (2023)	Temi	1*	1 (SS)
Shawler et al. (2024)	Farah	2	
Taylor (2020)	Herbert	1	1 (SS)
Thomas et al. (2022)	Linda	1	1 (SS)
Thomas et al. (2023)	Maude	1	1 (SS)
Thomas (2024)	P1	1	1 (SS)

*Note*: An (*) indicates that the authors described attempting a prior CSA application but did not provide the data, and thus the study was not included in our analysis. CSA = competing stimulus assessment; SS = stimulus set.

In total, 32 CSA validations across 17 studies and 25 participants were identified, but only 20 validations across 13 studies and 17 participants met our criteria for calculating predictive validity. Only two studies (Frank‐Crawford et al., 2024; Imler & Weyman, 2024) evaluated stimuli that did not meet the HC stimulus definition for a total of four applications. Specifically, Imler and Weyman (2024) compared the efficacy of short‐ and long‐latency stimuli (one of each per participant), and Frank‐Crawford et al. (2024) compared the efficacy of a high‐preference stimulus (that was not an HC stimulus) and an HC stimulus. However, Imler and Weyman changed the measurement of challenging behavior between the CSA application and validation (from latency to first response to responses per minute, respectively). Slightly more than half of the 32 validations (17, 53.13%) evaluated stimulus sets in which multiple HC stimuli were available. Maintenance data were available for seven of the 25 participants (28%) with validations.

#### Stimulus characteristics

A total of 738 stimuli were assessed across 87 applications. All 72 participants had at least one application that included leisure items. Three applications included an undefined attention trial, and one included three types of physical attention. Two applications included food trials.

#### CSA methods

##### General assessment procedures

Table 3 outlines the parameters for all 87 CSA applications included. Researchers developed CSAs in a variety of ways. Researchers commonly reviewed the results of preference assessments (41, 47.13%), obtained clinician nominations (37, 42.53%), reviewed the results of the *Reinforcer Assessment for Individuals with Severe Disabilities* (Fisher et al., 1996; 31, 35.63%), and hypothesized the sensory consequence of the behavior (24, 27.59%) when selecting stimuli to include in CSAs. The number of stimuli ranged from four to 16. Most test stimuli were categorized as leisure stimuli. Most CSA applications measured engagement (i.e., participants actively manipulating the stimulus) rather than contact (i.e., physically touching the stimulus with or without further manipulation) with test stimuli, but six of 87 (6.89%) only measured contact and nine (10.34%) measured both contact and engagement. Each trial within the CSA lasted between 1 and 10 min (*Mdn* = 4 min) and assessed test stimuli in one to six series (*Mdn* = 3 series). Researchers most frequently defined a HC stimulus as a stimulus that produced an 80% reduction in challenging behavior relative to the no‐stimulus control trial (63, 72.41%; see column titled “HCS Criteria”). Most applications implemented procedures that identified at least one HC stimulus (52, 59.77%), and 19 (21.84%) implemented procedures that identified at least one HC stimulus with disruption. Sixteen applications (18.39%) did not identify an HC stimulus or an HC stimulus with disruption.

**TABLE 3:** Parameters of CSA applications.

Citation	Participant	Selection basis	# of stimuli	Type of stimuli	Engagement or contact	Trial duration	# of Series	HCS criteria	CSA or A‐CSA	Mod.	Contingencies implemented
Dowdy et al. (2020)	Shane	PA	4	L	E	2 min	3	Unspecified	S	‐	‐
Falligant, Carver, et al. (2021)	Nick	Unspecified	4	L	E	135 s	3–4	Unspecified	*	‐	FR 1 A & RB
Falligant, Hardesty, et al. (2021)	Paolo	CI, CN (both)	6, 6	L	E	180 s–360 s	3	80% reduction	Aug. (P)	PE	‐
Frank‐Crawford et al. (2023)	P1	RAISD, PA, CN (H)	8	L	E	8 min	3	80% reduction	S	‐	‐
	P2	RAISD, PA, CN (H)	12	L	E	2 min	3	80% reduction	Aug. (P, RB + P)	‐	‐
	P3	RAISD, PA, CN (H)	7	L	E	5 min	3	80% reduction	S	RB other	‐
	P4	RAISD, PA, CN (H) (both)	12, 12	L	E	2 min, 2 min	3	80% reduction	S	PE	‐
	P5	RAISD, PA, CN (H)	9	L	E	7 min	3	80% reduction	S	RB other	‐
	P6	RAISD, PA, CN (H)	9	L	E	5 min	3	80% reduction	Aug. (P)	PE	‐
	P7	RAISD, PA, CN (H) (both)	5, 8	L	E	5 min, 5 min	3	80% reduction	S	PE	‐
	P8	RAISD, PA, CN (H)	7	L	E	2 min	3	80% reduction	S	PE	‐
	P9	RAISD, PA, CN (H)	8	L, A	E	4 min	3	80% reduction	Aug. (P, RB + P)	PE	‐
	P10	RAISD, PA, CN (H)	8	L	E	3 min	3	80% reduction	Aug. (P, RB + P)	PE, RB other	‐
	P11	RAISD, PA, CN (H)	12	L	E	8 min	3	80% reduction	S	RB other	‐
	P12	RAISD, PA, CN (H)	11	L	E	1 min	3	80% reduction	Aug. (P)	PE	‐
	P13	RAISD, PA, CN (H)	8	L	E	1 min	3	80% reduction	S	PE	‐
	P14	RAISD, PA, CN (H) (both)	7, 8	L, A	E	8 min, 4 min	3	80% reduction	Aug. (P, RB + P)	PE	‐
	P15	RAISD, PA, CN (H)	9	L, A	E	7 min	3	80% reduction	Aug. (P, RB + P)	PE	‐
	P16	RAISD, PA, CN (H)	11	L	E	3 min	3	80% reduction	S	PE	‐
Frank‐Crawford et al. (2024)	Winston	Unspecified	9	L	E	2 min	4	No elopement in 3 of 4 trials, mean engagement 80%	S	‐	FR 1 Tang.
Hagopian et al. (2020)	1	RAISD, CN	8	L	E	4 min	3	80% reduction	Aug. (P, RB + P)	‐	‐
	2	RAISD, CN	8	L	E	2 min	3	80% reduction	Aug. (P, RB + P)	‐	‐
	3	RAISD, CN	10	L	E	1 min	3	80% reduction	Aug. (P, RB + P)	‐	‐
	4	RAISD, CN	16	L	E	2 min	3	80% reduction	Aug. (P, RB + P)	‐	‐
	5	RAISD, CN	11	L	E	2 min	3	80% reduction	Aug. (P, RB + P)	‐	‐
	6	RAISD, CN	7	L	E	5 min	3	80% reduction	Aug. (P, RB + P)	‐	‐
Imler & Weyman (2024)	Tucker	(H), Consult, DO	7	L	E	5 min	3	Longest latency	S	‐	‐
	Tinley	(H), Consult, DO	7	L	E	5 min	3	Longest latency	S	‐	‐
	Walter	(H), Consult, DO	6	L	E	5 min	3	Longest latency	S	‐	‐
Jeglum et al. (2022)	Noelle	PA, DO (both)	8, 10	L	E	5 min, 6 min	1, 2	80% reduction	S	‐	‐
Laureano et al. (2023)	1	CI, PA, CN	11	L	E, C	10 min	3	80% reduction	S	‐	‐
	2	Unspecified (all)	8, 9, 11	L	E, C	5 min, 2 min, 5 min	3	80% reduction	S	‐	‐
	3	IA, DO	6	L	E	2.25 min	3	80% reduction	S	‐	FR 1 A
	9	CI, PA, CN (1st); (H) (2nd)	6, 8	L	E, C	2 min, 3 min	3	80% reduction	S	‐	‐
	10	CI, PA, CN	8	L	C	3 min	3	80% reduction	S	‐	FR 1 A
	11	Unspecified	8	L	C	5 min	4	80% reduction	S	‐	‐
	12	Unspecified	7	L	E	5 min	4	80% reduction	S	‐	‐
	13	CI, PA, CN (1st); DO, (H) (2nd)	13, 14	L, F	E, C	5 min	2	80% reduction	S	‐	‐
	15	Unspecified	7	L	E	2 min	3	80% reduction	S	‐	FR 1 A
	17	Unspecified	12	L	C	3 min	4	80% reduction	S	‐	‐
	19	CI, PA, CN	12	L	E	2 min	5	80% reduction	S	‐	FR 1 A
	20	Unspecified	8	L	E, C	3 min	5	80% reduction	S	‐	‐
	21	CI, PA, CN	12	L	E	4 min	3	80% reduction	S	‐	‐
	24	Unspecified	6	L	E	2 min	3	80% reduction	S	‐	FR 1 A
	25	Unspecified	11	L	C	5 min	3	80% reduction	S	‐	‐
	26	Unspecified	10	L	E	5 min	3	80% reduction	S	‐	‐
	30	Unspecified (both)	12, 9	L	C	5 min	6, 3	80% reduction	S	‐	‐
	31	Unspecified (all)	8, 8, 8	L	E	3 min, 7 min, 7 min	3	80% reduction	S	‐	‐
	32	Unspecified	8	L	E	5 min	4	80% reduction	S	‐	‐
	33	CI, PA, CN (both)	7, 7	L	E	10 min	3	80% reduction	S	‐	‐
	34	CI, PA, CN	15	L	E	3 min	3	80% reduction	S	‐	‐
Leif et al. (2020)	Julia	TI	7	L	E	3 min	3	75% engagement, less than 15% challenging behavior	Aug. (P)	DRA	‐
	Doug	TI	8	L	E	3 min	3	75% engagement, less than 15% challenging behavior	Aug. (P)	DRA	‐
	Tammy	TI	8	L	E	3 min	3	75% engagement, less than 15% challenging behavior	Aug. (P)	DRA	‐
	Mark	TI	8	L	E	3 min	3	75% engagement, less than 15% challenging	Aug. (P)	DRA	‐
Miller et al. (2022)	Brian	RAISD	6	L	E	5 min	2–3	Unspecified	S	‐	FR 1 Tang.
	Mark	RAISD	8	L	E	5 min	2–3	Unspecified	S	‐	FR 1 Tang.
	Wayne	RAISD	9	L	E	5 min	2–3	Unspecified	S	VP	FT Tang. & EXT
	Keith	RAISD	6	L	E	5 min	2–3	Unspecified	S	‐	FR 1 Tang.
Newcomb et al. (2019)	Ted	CI, Safety	4	L, Phys	E	3 min	3	Unspecified	S	‐	FR 1 RC
Rosenzweig et al. (2024)	Brian	PA	9	L	E	5 min	3	80% reduction	S	‐	‐
	Daniel	PA	7	L	E	6 min	3	80% reduction	Aug. (P, RB + P)	‐	‐
	Amy	PA	7	L	E	7 min	3	80% reduction	Aug. (P, RB + P)	‐	‐
	Seth	PA	7	L	E	8 min	3	80% reduction	S	‐	‐
Ruckle et al. (2023)	Patrick	PA	7	L	E	3 min	3	80% reduction in free access	Aug. (P, RB + P)	‐	‐
Schmidt et al. (2021)	Jeb	CI, DO, PA (1st); CI, DO (2nd)	14, 8	L	E	2 min	Unspecified	80% reduction excluding RB	Aug. (P, RB + P)	‐	‐
Shawler et al. (2023)	Temi	Unspecified	8	L	E	2–3 min	3	80% reduction	Aug. (P)	‐	‐
Shawler et al. (2024)	Farah	CI, Ease, PA (both)	5, 5	L	E	2 min	6	Lower than alone condition in FA	S	‐	EXT
Taylor (2020)	Herbert	Unspecified	6	L, F	E	3 min	3	Low rates of pica, high engagement	S	‐	‐
Thomas et al. (2022)	Linda	RAISD, CI, CN	8	L	E	5 min	3	80% reduction, 50% engagement	S	‐	‐
Thomas et al. (2023)	Maude	PA, CI	5	L	E	8 min	3	95% reduction or near‐zero levels	Aug. (P, RB + P)	‐	‐
Thomas (2024)	P1	RAISD, PA, DO	11	L	E	2 min	3	80% reduction, 50% engagement	S	‐	‐

*Note*: Some participants experienced response blocking or disruption via protective equipment that was applied noncontingently prior to the session. The “Contingencies implemented” only refers to contingencies implemented by therapists within the trial and does not include noncontingent procedural elements. # = number; (1st) = applies to the first application; (2nd) = applies to the second application; A = attention; A‐CSA = augmented competing stimulus assessment; (all) = applies to all applications; Aug. = augmented; (both) = applies to both applications; C = contact; CI = caregiver interview; CN = clinician nomination; CSA = competing stimulus assessment; DO = direct observation; DRA = differential reinforcement of alternative behavior; E = engagement; Ease = ease of delivery; EXT = extinction; F = food; FA = functional analysis; FR = fixed ratio; FT = fixed‐time delivery; (H) = hypothesized sensory consequence; L = leisure items; Mod. = modifications; P = prompting; PA = preference assessment or otherwise reported as preferred; PE = protective equipment; Phys = physical attention; RAISD = *Reinforcer Assessment for Individuals with Severe Disabilities*; RB = response blocking; RB other = blocking another behavior; RC = response cost; S = standard; Tang. = tangible; TI = teacher interview; VP = verbal praise.

* indicates the procedures did not meet either of our definitions for standard or augmented CSAs.

##### Assessment classification

Most applications (59, 67.82%) followed standard CSA procedures, and 27 (31.03%) met the A‐CSA definition. All A‐CSAs targeted automatically maintained behavior. One application (1.15%) used response blocking but did not include a free‐access condition, thus not meeting either definition, so it was not included in the data analysis. All A‐CSAs used prompts, and 18 (66.67%) used response blocking with prompting for stimulus engagement. It is important to note that three (16.67%) of these response‐blocking procedures entailed noncontingent application of protective equipment rather than contingent blocking by a therapist to either mitigate risk of injury or more effectively promote redirection to stimulus engagement (Frank‐Crawford et al., 2023). Other modifications were made to 24 (27.59%) of all CSA applications. These included protective equipment (16, 66.67%), differential reinforcement of alternative behavior (4, 16.67%), blocking alternative challenging behavior (4, 16.67%), and verbal praise (1, 4.17%). Across all CSAs, experimenters provided only the test stimuli.

##### Contingencies implemented

For 11 (78.57%) applications targeting socially reinforced challenging behavior, experimenters explicitly arranged CSA conditions similar to the relevant test condition of the functional analysis (during all test and control trials). For CSAs targeting challenging behavior maintained by attention, preferred attention was withheld at the start of the session and delivered briefly following each instance of target challenging behavior and then removed again. When targeting challenging behavior maintained by access to tangibles, the preferred item was removed at the start of the session and delivered briefly following each instance of target challenging behavior and then removed again. Of these, 10 (90.90%) entailed continuous schedules of reinforcement and one (9.09%) consisted of fixed‐time delivery. One application for socially reinforced behavior included a response‐cost procedure for an attention function (Newcomb et al., 2019). The response‐cost procedure involved the therapist terminating physical stimulation that was provided according to each test condition and moving away from the participant contingent on aggression. The remaining applications for socially reinforced behavior programmed extinction.

#### CSA validation methods

Table 4 outlines the validation methods coded (columns) per participant (rows). Researchers validated 64 confirmed stimuli, four of which were not indicated in the corresponding CSA application. Five CSA applications (5.75%) evaluated consistency across repeated administrations for at least a subset of test stimuli. Sixteen validations (50%) collected engagement data across eight studies. Four validations (12.50%) within one study measured both contact and engagement. Across 32 validations, seven (21.88%) used a schedule‐thinning arrangement and three (9.38%) included terminal‐probe arrangements. Most validations (24, 75%) evaluated HC stimuli within a larger treatment package. Treatment packages for socially reinforced behavior (*n* = 6) often included functional communication training (4, 66.67%) and either response blocking or extinction (6, 100%). Note that the authors did not specify whether response blocking in this context was paired with prompted engagement for these applications. Treatment packages for automatically maintained behavior (*n* = 19) frequently included prompting engagement with identified HC stimuli (16, 84.21%) and some form of response blocking with prompted engagement (with the exception of the response‐blocking‐only evaluation for Jeb; Schmidt et al., 2021), protective equipment, or redirection to either engage with identified HC stimuli or discard pica items (11, 57.89%). Specifically, 15 of 19 applications (78.95%) programmed prompted engagement to occur independently of programmed response blocking or redirection contingencies, two (10.53%) programmed response blocking that was not followed by prompted engagement, one (5.26%) programmed redirection that did not entail prompted engagement with HC stimuli, and six (31.58%) programmed a combination of these procedures. Note that five of the 19 treatment packages included prompted engagement both with and without response blocking or redirection. Thomas et al. (2023) was the only treatment package to include a response‐cost procedure. Four of the 32 validations (12.50%) included noncontingent access to other preferred stimuli, three of which targeted automatically maintained behavior and one which targeted a mixed function.

**TABLE 4:** Parameters of validations and evaluations of consistency.

Citation	Participant	Function	# of stimuli	Evaluation of consistency or validation	Engagement or contact	Nonindicated stimulus included	Schedule thinning	Terminal probe	Treatment package	Other Preferred Stimuli	MT Data
Dowdy et al. (2020)	Shane	Auto	1	V	E	‐	‐	‐	‐	‐	X
Falligant, Carver, et al. (2021)	Nick	Ambiguous/Attn	4	V	‐	‐	‐	‐	FT A & RB	‐	‐
Falligant, Hardesty, et al. (2021)	Paolo	Auto	4	V	E	‐	‐	‐	FT A, P, & PE	‐	‐
Frank‐Crawford et al. (2023)	P2	Auto	12	Consistent	‐	‐	‐	‐	‐	‐	‐
	P6	Auto	7	Consistent	‐	‐	‐	‐	‐	‐	‐
	P9	Auto	2	Consistent	‐	‐	‐	‐	‐	‐	‐
	P10	Auto	1	Consistent	‐	‐	‐	‐	‐	‐	‐
Frank‐Crawford et al. (2024)	Winston	Tang	2	V	E	X	X	X	FCT & RB	‐	‐
Imler & Weyman (2024)	Tucker	Auto	2	V	‐	X	‐	‐	‐	‐	‐
	Tinley	Auto	2	V	‐	X	‐	‐	‐	‐	‐
	Walter	Auto	2	V	‐	X	‐	‐	RB + P	‐	‐
Jeglum et al. (2022)	Noelle	Auto	2	V	E	‐	‐	‐	P & RB + P	X	X
Leif et al. (2020)	Julia	Auto	1	V	E	‐	X	‐	DRA & P	‐	‐
	Doug	Auto	2	V	E	‐	X	‐	DRA & P	‐	‐
	Tammy	Auto	1	V	E	‐	X	‐	DRA & P	‐	‐
Miller et al. (2022)	Brian	Esc, Tang	1	V	‐	‐	X	‐	EXT, FCT, & Mult.	‐	‐
	Wayne	Esc, Tang, Attn	1	V	‐	‐	X	X	EXT, FCT, & Mult.	‐	‐
Newcomb et al. (2019)	Ted	Attn	3	V	‐	‐	‐	‐	RB + P	‐	X
Rosenzweig et al. (2024)	Brian	Auto	3	V	E, C	‐	‐	‐	P & Rotate	‐	‐
	Daniel	Auto	3	V	E, C	‐	‐	‐	P & Rotate	‐	‐
	Amy	Auto	5	V	E, C	‐	‐	‐	P & Rotate	‐	‐
	Seth	Auto	6	V	E, C	‐	‐	‐	P & Rotate	‐	‐
Ruckle et al. (2023)	Patrick	Auto	2	Both	‐	‐	X	‐	DRA, FT Token, & RIRD + P	‐	‐
Schmidt et al. (2021)	Jeb	Auto	4	V	E	‐	‐	‐	P & RB + P (except RB‐only condition)	‐	‐
Shawler et al. (2023)	Temi	Auto	3	V	E	‐	‐	‐	DRA, FCT, Mult., P, & RB + P	‐	‐
Taylor (2020)	Herbert	Auto	Unspec.	V	‐	‐	‐	‐	DRA & RIRD	‐	X
Thomas et al. (2022)	Linda	Auto	5	V	‐	‐	‐	X	‐	X	X
Thomas et al. (2023)	Maude	Auto	2	V	‐	‐	‐	‐	P, RC, & RIRD + P	X	X
Thomas (2024)	P1	Auto, Tang	3	V	‐	‐	‐	‐	‐	X	X

*Note*: The “MT Data” column only refers to maintenance data taken in the context of a validation. An “X” indicates that the validation included the relevant procedures. A = attention; C = contact; Consistent = evaluation of consistency; CSA = competing stimulus assessment; DRA = differential reinforcement of alternative behavior; E = engagement; EXT = extinction; FCT = functional communication training; FT = fixed‐time delivery; HCS = high‐competition stimuli; MT = maintenance data; Mult. = multiple schedule; P = prompting; PE = protective equipment; RB = response blocking; RC = response cost; RIRD = response interruption and redirection; Rotate = stimulus rotation; Unspec. = Unspecified; V = validation.

### Data analysis

#### Correlation between percentage of reduction and engagement

**FIGURE 2:**
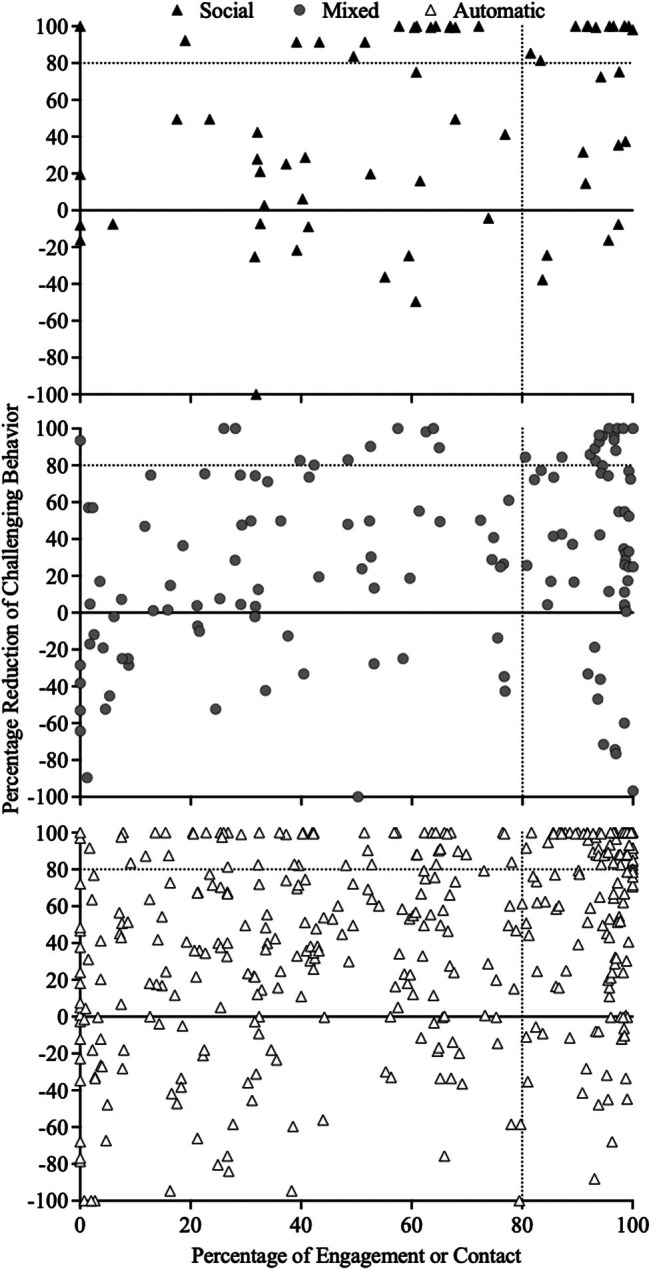
Percentage of engagement or contact and percentage of reduction in challenging behavior (CSAs). Data are grouped by function of behavior. The dotted horizontal line indicates an 80% reduction in challenging behavior, and the dotted vertical line indicates 80% engagement or contact.

Figure 2 depicts the relation between percentage of engagement or contact and the percentage of reduction for all test stimuli in standard CSAs. Negative values indicate that challenging behavior increased during test conditions when compared with the control trials. Table 5 displays the stimuli that produced more than a 100% increase in challenging behavior relative to a no‐stimulus control. Across groups, higher engagement occasionally correlated with higher reductions in challenging behavior, although this was not always the case. For example, in the automatic group (bottom panel), several stimuli produced complete reductions of challenging behavior with minimal engagement or contact. Interestingly, 29 (32.22%) stimuli in the social group, 84 (65.63%) in the mixed group, and 141 (27.70%) in the automatic group produced an increase in challenging behavior. In some cases, engagement with these stimuli was high despite the increase in challenging behavior.

**TABLE 5:** Percentage of reduction values outside of Figure 2 axes.

Social		Mixed		Automatic	
Percentage of engagement	Percentage of reduction	Percentage of engagement	Percentage of reduction	Percentage of engagement	Percentage of reduction
1.73	−277.57	0	−3,178.83	0	−145.14
13.00	−553.66	0	−2,001.23	0	−480.16
16.00	−224.39	0	−502.46	0	−668.92
20.79	−233.43	1.35	−154.53	1.58	−172.46
26.23	−189.29	1.51	−448.93	2.42	−295.04
28.00	−103.16	2.56	−315.11	2.42	−110.08
30.45	−277.57	2.7	−127.76	4.84	−195.04
59.41	−321.71	5.41	−181.29	7.43	−189.29
69.31	−498.29	9.13	−2,911.19	7.50	−170.92
70.05	−233.43	13.88	−1,706.83	16.15	−322.39
77.97	−145.14	24.54	−1,439.19	18.01	−155.22
89.00	−395.12	25.23	−181.29	19.79	−201.72
99.00	−321.71	39.04	−181.29	20.05	−189.29
		44.31	−502.45	22.29	−101.15
		46.89	−1,251.84	22.93	−122.82
		47.45	−208.06	23.44	−364.71
		51.23	−877.15	24.82	−229.41
		52.11	−181.29	24.99	−164.96
		52.27	−609.51	27.23	−129.41
		52.86	−341.87	29.81	−177.61
		53.47	−582.75	31.97	−161.76
		55.55	−127.76	32.18	−189.29
		55.59	−1,010.96	34.65	−1028.00
		56.61	−288.34	34.69	−974.19
		62.47	−341.87	39.69	−2919.35
		63.56	−1,171.55	40.09	−3146.86
		65.16	−101.00	40.43	−264.00
		65.66	−1,037.73	45.05	−321.71
		69.83	−341.87	47.58	−225.12
		74.48	−261.58	50.18	−194.83
		76.15	−770.09	50.49	−233.43
		83.04	−234.82	51.24	−143.10
		86.35	−422.15	51.49	−145.14
		86.94	−181.29	52.42	−305.12
		87.11	−689.80	55.37	−103.03
		88.59	−234.82	59.01	−122.41
		88.89	−101.00	59.41	−145.14
		89.64	−127.76	64.36	−365.86
		91.59	−154.53	68.56	−189.29
		95.18	−3,045.02	71.02	−110.34
		96.25	−234.82	71.53	−145.14
		96.69	−127.76	72.64	−158.00
		96.71	−475.69	78.09	−217.24
		97.03	−823.62	78.96	−233.23
		97.77	−636.28	81.93	−145.14
		97.91	−341.87	85.39	−233.43
		98.82	−529.22	86.34	−213.14
		99.24	−101.00	86.48	−341.18
				89.52	−105.12
				92.01	−164.65
				98.51	−145.14
				99.06	−170.92
				99.26	−189.29

**FIGURE 3:**
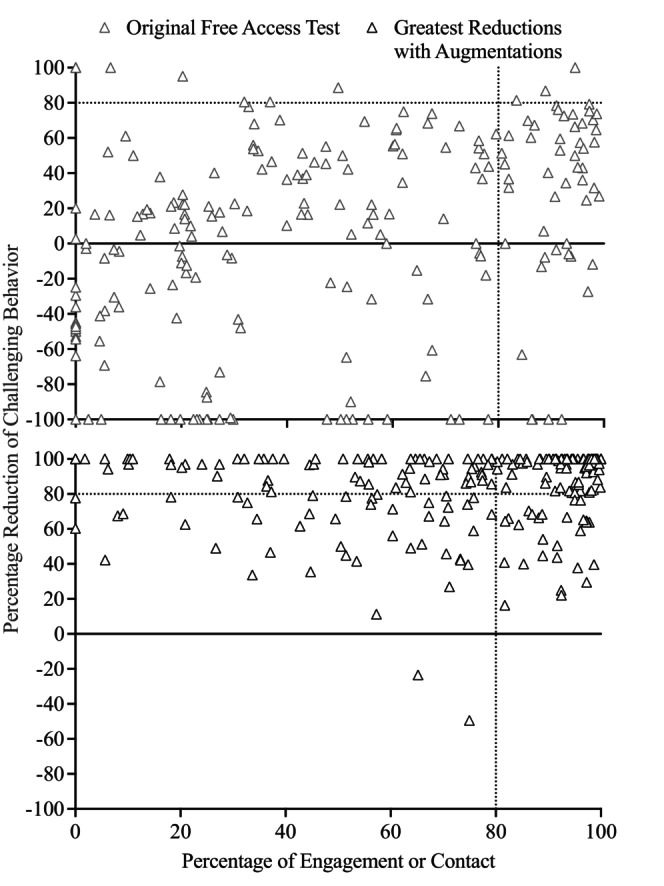
Percentage of engagement or contact and percentage of reduction in challenging behavior (A‐CSAs). The dotted horizontal line indicates an 80% reduction in challenging behavior, and the dotted vertical line indicates 80% engagement or contact.

Figure 3 depicts the relation between engagement and reductions in challenging behavior for the free‐access test (top panel) and the augmented condition that produced the greatest percentage of reduction (bottom panel) for A‐CSAs. When comparing both panels, augmentations often produced greater engagement and reductions in challenging behavior. However, some stimuli produced large reductions in the absence of high engagement. Notably, only two stimuli produced an increase in challenging behavior when examining the most efficacious augmented conditions.

#### Percentage of HC stimuli identified

**FIGURE 4:**
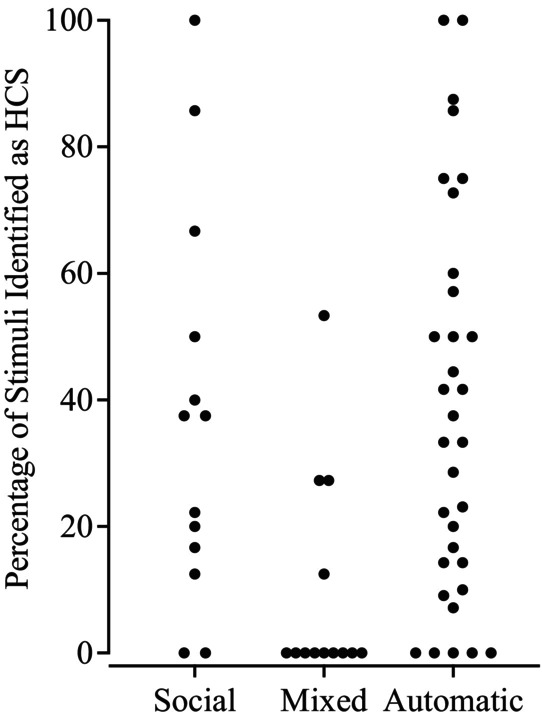
Percentage of stimuli identified as HC stimuli in CSAs. Data are grouped by function of behavior and only represent CSA applications that did not include subsequent augmented conditions. CSA = competing stimulus assessment; HCS = high‐competition stimulus.

**FIGURE 5:**
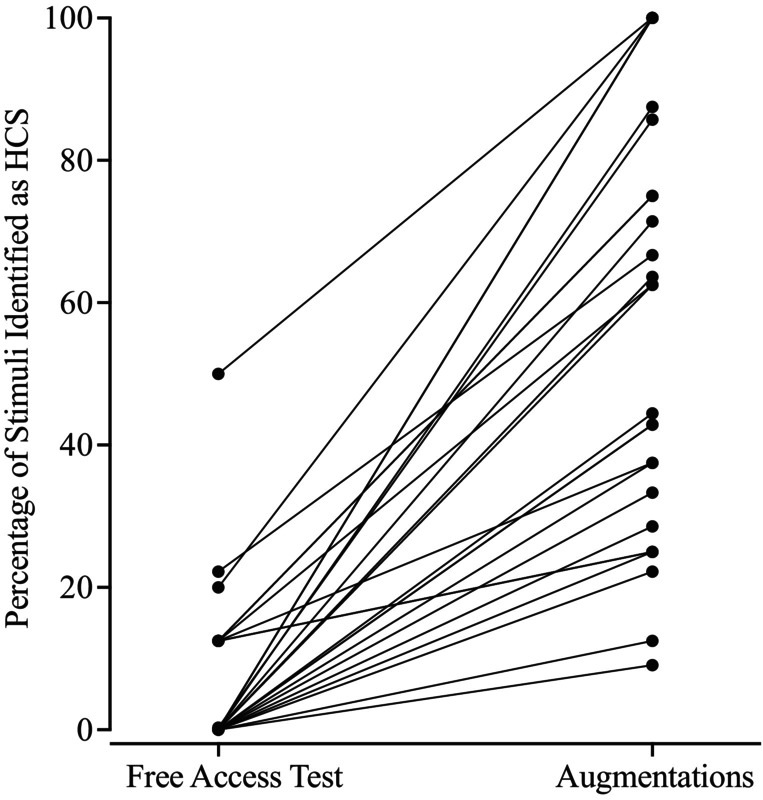
Percentage of stimuli identified as HC stimuli in augmented competing stimulus assessments (A‐CSAs). Data on the left represent the results of the free‐access test, and data on the right represent the results of the augmented condition that identified the greatest percentage of HC stimuli. All A‐CSA applications targeted automatically maintained challenging behavior. HCS = high‐competition stimulus.

Figure 4 represents the percentage of HC stimuli identified per application within each behavioral function among standard CSAs. Within each group, individual applications demonstrate a wide range of HC stimuli identified. The mixed group demonstrated the least variability, although it is worth noting that most of the mixed applications came from Laureano et al. (2023), which did not include augmented procedures. Figure 5 shows the results of the free‐access test and the augmented conditions that yielded the greatest percentage of HC stimuli within A‐CSAs. Recall that all A‐CSA applications meeting our definition targeted automatically maintained challenging behavior. Augmentations always resulted in the identification of a greater percentage of HC stimuli. In the automatic group, 219 of the 509 (43.03%) stimuli tested were identified as HC stimuli. In the socially reinforced group, 28 of 90 (31.11%) stimuli tested were identified as HC stimuli. In the mixed group, 13 of the 128 (10.16%) stimuli tested were identified as HC stimuli.

#### Predictive validity correspondence

**FIGURE 6:**
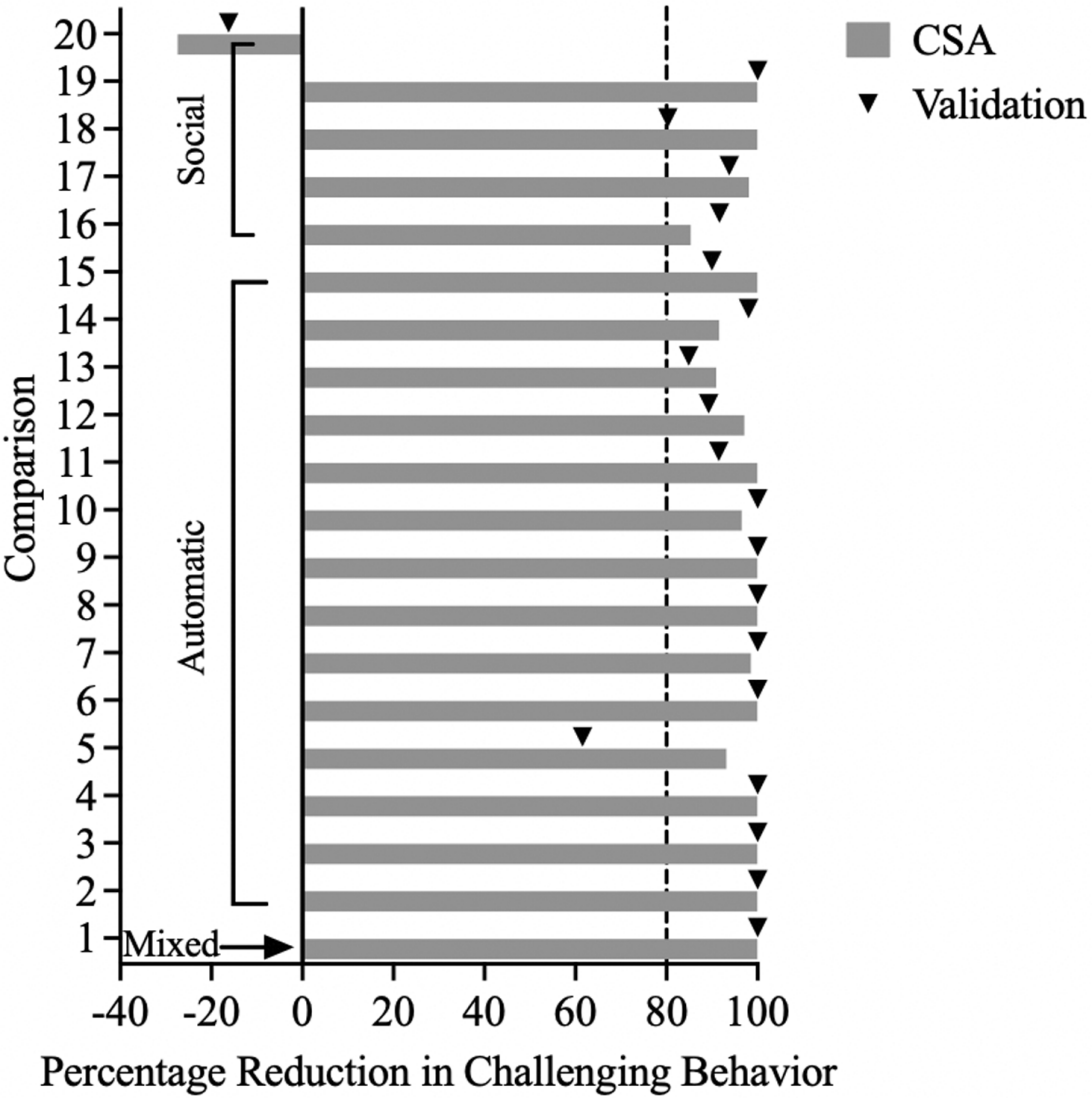
Percentage of reduction in challenging behavior during CSA and corresponding validation. The dashed vertical line represents an 80% reduction in challenging behavior. CSA = competing stimulus assessment.

Figure 6 shows the percentage of reduction in challenging behavior during the CSA application and corresponding validation. Similar to Haddock and Hagopian (2020), we compared the highest percentage of reduction within the CSA with the validation, and stimulus sets counted as a single validation. We calculated predictive validity for 19 identified HC stimuli or stimulus sets and one stimulus not indicated by the assessment. Seventeen HC stimulus validations (89.47%) confirmed the results of the CSA; the sole stimulus that was not indicated by the CSA performed similarly in the validation as well. The sole validation from the mixed group, three of four (75%) validations from the social group, and 13 of the 14 (92.86%) validations for the automatic group demonstrated predictive validity. Of note, the sole validation from the social group that did not meet our criteria for predictive validity correspondence (i.e., a difference of 10 percentage points or fewer) still demonstrated an 80% reduction in challenging behavior.

## DISCUSSION

Results of the current review indicate that within published data sets, CSAs and A‐CSAs continue to identify HC stimuli when applied to challenging behavior maintained by various functions. Further, the predictive validity of these assessments appears high when researchers provide identified HC stimuli individually or as part of a treatment package; however, this is based on 17 of 19 predictive validity calculations, where only seven validations incorporated schedule‐thinning procedures, so these findings require additional investigation. As in Haddock and Hagopian (2020), CSA applications largely targeted automatically maintained behavior, but the number of applications for socially reinforced behavior has increased in recent years. Of note, attention was the most common social reinforcer maintaining challenging behavior. Two CSA applications for behavior maintained by access to attention in the current review tested the competition effects of different attention topographies. This poses interesting questions for future researchers concerning the parameters of attention serving as functional reinforcers and whether different topographies of attention compete with one another. Moreover, evaluating different topographies of attention might be conceptualized as attempts to “match” stimuli to those delivered in the natural environment, suggesting the potential for matching procedures extending to CSAs and A‐CSAs for socially reinforced behavior. Additional discussion on this topic can be found in the Applications for socially reinforced challenging behavior section below.

The underlying mechanism responsible for reinforcer competition also warrants additional investigation. As with Haddock and Hagopian (2020) and Laureano et al. (2023), the phenomenon in which stimuli produce reduced levels of challenging behavior without simultaneously producing high levels of engagement was also observed in the current review. This lack of a consistent positive correlation suggests that (a) competition may not be dependent on stimulus engagement or that (b) motivation for both challenging behavior and stimulus engagement may fluctuate within the assessment. The current review found that the predictive validity of CSAs is high in extended analysis, so perhaps validations comparing performance of high‐ and low‐engagement HC stimuli will help answer these questions. Obtaining intrasubject replications via repeated testing of stimuli across days may shed light on the questions of satiation and fluctuations in overall response level within CSAs as well. Specifying the time between tests would further illuminate the role of response variability in CSA results.

### Applications for socially reinforced challenging behavior

Another consideration highlighted by this and previous reviews is that CSA applications for socially reinforced behavior appear to yield a moderate proportion of HC stimuli (Haddock & Hagopian, 2020; Laureano et al., 2023). Results of the current review more closely align with those of Laureano et al. (2023), in which 30.80% of stimuli tested for competition with socially reinforced behavior proved efficacious. Despite the increase in applications for socially reinforced behavior, these applications still represent a small sample of the published CSA literature. Further, the current literature offers little guidance to researchers and clinicians looking to modify CSA procedures when identifying HC stimuli for socially reinforced behavior. The one example of augmented procedures (i.e., response blocking without prompted engagement) for socially reinforced challenging behavior in the current review did not include a free‐access test like in A‐CSAs for automatically maintained behavior. Thus, we were unable to compare the results across standard and augmented CSA procedures for this application.

The limited applications of CSAs for socially reinforced behavior stand in stark opposition to the prevalence of social functions for challenging behavior at large. In their review of the functional analysis literature, Melanson and Fahmie (2023) found that most outcomes indicated a social function for challenging behavior. Specifically, 71.35% of the included single‐function outcomes indicated a social function and only 4.20% of the multiple‐control outcomes indicated that challenging behavior was partially automatically maintained. This discrepancy between general prevalence and CSA applications suggests that the technology, as it relates to socially reinforced behavior, is lagging behind that for automatically maintained behavior. Therefore, it would behoove researchers to investigate CSA augmentations and the efficacy of HC stimuli for socially reinforced challenging behavior. Continued demonstrations of HC stimuli supporting treatment goals for socially reinforced challenging behavior may inform clinical practice going forward.

One underlying factor influencing the development of augmentations for socially reinforced behavior may be that CSA procedures varied across studies. For example, the current review found that researchers did not always arrange continuous schedules of reinforcement for socially reinforced challenging behavior and one application implemented a response‐cost procedure instead. Moreover, CSA applications for behavior maintained by mixed reinforcement often do not program schedules of reinforcement for challenging behavior (e.g., Laureano et al., 2023). Future researchers may consider which A‐CSA procedures are relevant for applications of socially reinforced behavior and develop modifications specific to these applications. The CSA for tasks (Schmidt et al., 2021) may be of particular interest when identifying HC tasks for socially reinforced behavior; providing an individual with a task that can be lengthened or shortened depending on the scheduled delay to reinforcement may compete with repetitive mands and challenging behavior more effectively and be viewed more favorably by caregivers, but this remains to be seen.

### Assessment prescriptiveness and HC stimulus durability

The current review suggests that published CSA validations wherein the effects of HC stimuli or contingencies from the CSA or A‐CSA can be isolated and quantified are increasing. Caregivers implemented several validations (e.g., Jeglum et al., 2022; Shawler et al., 2023; Thomas et al., 2023). Moreover, although not included in our formal analysis, a cursory review of the data indicated that 20 of the total 32 validations used HC stimuli or HC stimuli with disruption that were associated with an objective measure for reduction in challenging behavior in the CSA. Most of these validations used stimuli that were associated with at least an 80% reduction in challenging behavior relative to the no‐stimulus control in the CSA. Note that some of these validations also included a prespecified criterion for engagement. Some validations involved simultaneous or rotating presentation of multiple HC stimuli or HC stimuli with disruption within a given treatment session. Whether stimuli should be selected for treatment validation based on engagement remains unclear given the findings that reductions in challenging behavior may occur without corresponding increases in engagement. However, because the primary goal of treatment is to reduce challenging behavior, stimuli should be selected based on their ability to produce at least an 80% reduction in challenging behavior relative to the no‐stimulus control condition in the CSA.

Our calculations, like Haddock and Hagopian (2020), verified that HC stimuli often perform similarly across the CSA and subsequent validation (*n* = 17 of 19). Given most validations consisted of a treatment package (*n* = 24), future researchers may consider evaluating consistency across repeated CSA administrations to allow for repeated efficacy measures of HC stimuli in the absence of other contingencies that may suppress challenging behavior. Similarly, maintenance data on HC stimulus efficacy, either in isolation or within the treatment context, remain important to determine the durability of treatment components over time. Regarding the potential for false‐negative results, few validations in the current review tested stimuli that a CSA indicated do not compete with challenging behavior (*n* = 4). As a result, the true extent of CSA prescriptiveness remains unclear. Along these lines, it is important to note that this review encompasses only published CSA and A‐CSA data. Additional CCCS, such as Laureano et al. (2023), are critical for obtaining measures of CSA and A‐CSA effectiveness that are minimally influenced by publication and selection bias. Most published analyses appear to be retrospective CCCS. While useful for evaluating historical data on CSA efficacy, there were likely many uncontrolled variables that influenced the outcomes of these analyses. Future researchers should prioritize conducting prospective analyses to better standardize participant and procedural characteristics and, hopefully, bolster claims about the effectiveness of CSAs and A‐CSAs.

Future researchers may consider evaluating probes for terminal schedules of reinforcement when electing to use HC stimuli within the context of schedule thinning. Miller et al. (2022) provides one potential model for future researchers. Their procedures entailed first conducting a progressive interval assessment, which consisted of exposing participants to successively longer extinction intervals to identify the point at which challenging behavior began to increase. These data were then used to identify an initial schedule‐thinning step by selecting the interval immediately preceding that associated with increased challenging behavior. During the validation, they probed the terminal schedule and only implemented progressive schedule thinning following a treatment breakdown. Alternatively, researchers may elect to implement terminal probe schedule thinning (e.g., Strohmeier et al., 2024, 2025) in which schedule thinning steps are derived from the latency to clinically significant levels of challenging behavior within the extinction component of a terminal schedule probe. In the case of HC stimulus applications for socially reinforced behavior, terminal probes paired with extinction for challenging behavior may enhance the efficiency of such procedures. Moreover, conducting additional terminal probes could help quantify the proportion of cases wherein the inclusion of HC stimuli enabled more rapid schedule thinning. The current review found that only three validations included terminal schedule probes, so further evaluating the efficacy of HC stimuli during terminal probes remains warranted.

Relatedly, the current review highlights the changes in reinforcement contingencies that often occur when transitioning from CSAs to validations. For example, challenging behavior may be reinforced on a continuous schedule during the CSA before being placed on extinction during treatment. Functional communication training, differential reinforcement of alternative behavior, prompting, and response blocking combined with prompted engagement were common components of the validated treatment packages. These procedures necessarily shift reinforcement from challenging behavior to alternative responses not available to the participant during the CSA. We could not calculate predictive validity of HC stimuli in isolation for all the validations described due to the specific treatment phases arranged; furthermore, the inclusion of multiple treatment components poses challenges for determining the isolated effects of HC stimuli. Future researchers should investigate how changes to reinforcement contingencies, with and without HC stimuli, may facilitate or hinder predictive validity between assessment and treatment data. Perhaps researchers can develop other means of aligning the assessment context with the treatment context as well.

Future researchers may also consider directly comparing the efficacy of stimulus sets and a single HC stimulus. Within the current review, 17 validations presented a stimulus set, rotated stimuli within the session, or did not specify the HC stimuli used across sessions. These arrangements offer advantages from a clinical utility perspective (e.g., may prevent satiation with a stimulus), although they make it difficult to interpret the predictive validity of a CSA across multiple identified HC stimuli. Future researchers may consider conducting validations of stimuli not indicated by the CSA by evaluating consistency across repeated administrations to rule out false‐negative results.

### Summary

The findings of the current review generally replicated and extended the findings reported by Haddock and Hagopian (2020). The current review found that published CSA applications for socially reinforced behavior and CSA validations across behavioral functions are increasing. Moreover, CSAs in the published literature often identify HC stimuli that continue to suppress challenging behavior during validations where treatment is evaluated over extended sessions. Outcomes of these applications and validations provide additional support for the use of CSAs to identify HC stimuli to employ as a component in the treatment of socially reinforced challenging behavior. This could be important, as socially reinforced challenging behavior continues to comprise the majority of functional analysis outcomes (Melanson & Fahmie, 2023). Further, several applications of A‐CSAs demonstrate that augmentations enhance the identification of HC stimuli for automatically maintained behavior. However, the variation in assessment procedures for applications for socially reinforced behavior and validations across all behavioral functions warrants additional investigation. Additional tests of CSA reliability and HC stimulus durability are also warranted. These developments pose several questions for future researchers interested in the mechanism and validation of reinforcer competition.

## AUTHOR CONTRIBUTIONS

All authors contributed to the conceptualization, methodology, and review and editing of the manuscript. The first and second authors participated in data curation and investigation and contributed to the original draft of the manuscript. The first author contributed formal analysis of the data and visualized data for the manuscript.

## CONFLICT OF INTEREST STATEMENT

There is no conflict of interest to declare.

## ETHICS APPROVAL

Approval from an institutional review board was not necessary for this review.

## Supporting information


**Table S1** *Databases, Database Hosts, and Search Terms*.

## Data Availability

Data that support the findings of this study are available on request from the corresponding author. Supporting information includes the databases, database hosts, and search terms for the literature search conducted in this study.

## Footnotes

^1^
Haddock and Hagopian (2020) used the term “validation” to describe treatment evaluations and extended analyses of HC stimuli. In cases where multiple stimuli were evaluated independently, these were considered separate validations. We use the term validation in the same manner throughout this review (see Number of CSA applications and validations section below for additional detail).

^2^
Schmidt et al. (2021) referred to the A‐CSA to identify competing tasks as a “competing task assessment.”
