# Supplementary material for: Recent developments and future directions for competing stimulus assessments
Source: J Appl Behav Anal. 2026 Jul 29;59(4):e70074. doi: 10.1002/jaba.70074 (PMC13416802; doi:10.1002/jaba.70074)
Supplement: Supplementary file 1 — Table S1 Databases, Database Hosts, and Search Terms. [file JABA-59-0-s001.docx]

**Table S1**

*Databases, Database Hosts, and Search Terms.*

| Database | Host | Search terms | Limits |
| --- | --- | --- | --- |
| Eric | EBSCOhost | (("competing items" OR "competing stimulus" OR "competing item" OR "competing stimuli" OR “competing task” OR “competing tasks”) AND (behavior* OR behaviour*)) OR (("preference assessment" OR "preference assessments") AND ("Problem Behavior" OR "problem behavior" OR "problem behaviors" OR "destructive behavior" OR "destructive behaviors" OR “maladaptive behavior” OR “maladaptive behaviors” OR “challenging behavior” OR “challenging behaviors”)) | 2019–2024; Academic Journals |
| PsycINFO, PsycARTICLES* | Ovid | ((("competing items" or "competing stimulus" or "competing item" or "competing stimuli" or "competing task" or "competing tasks") and (behavior* or behaviour*)) or (("preference assessment" or "preference assessments") and ("Problem Behavior" or "problem behavior" or "problem behaviors" or "destructive behavior" or "destructive behaviors" or "maladaptive behavior" or "maladaptive behaviors" or "challenging behavior" or "challenging behaviors"))).ab,ti,tw. | 2019–2024; Peer-Reviewed Journals |
| Embase | Elsevier | (("competing items":ti,ab,kw OR "competing stimulus":ti,ab,kw OR "competing item":ti,ab,kw OR "competing stimuli":ti,ab,kw OR “competing task”:ti,ab,kw OR “competing tasks”:ti,ab,kw) AND (behavior*:ti,ab,kw OR behaviour*:ti,ab,kw)) OR (("preference assessment":ti,ab,kw OR "preference assessments":ti,ab,kw) AND ("Problem Behavior":ti,ab,kw OR "problem behavior":ti,ab,kw OR "problem behaviors":ti,ab,kw OR "destructive behavior":ti,ab,kw OR "destructive behaviors":ti,ab,kw OR “maladaptive behavior":ti,ab,kw OR “maladaptive behaviors”:ti,ab,kw OR “challenging behavior”:ti,ab,kw OR “challenging behaviors”:ti,ab,kw)) | 2019–2024; article, article in press, preprint |
| PubMed | PubMed.gov | (("competing items"[tw] OR "competing stimulus"[tw] OR "competing item"[tw] OR "competing stimuli"[tw] OR “competing task”[tw] OR “competing tasks”[tw]) AND (behavior*[tw] OR behaviour*[tw])) OR (("preference assessment"[tw] OR "preference assessments"[tw]) AND ("Problem Behavior"[Mesh] OR "problem behavior"[tw] OR "problem behaviors"[tw] OR "destructive behavior"[tw] OR "destructive behaviors"[tw] OR “maladaptive behavior”[tw] OR “maladaptive behaviors”[tw] OR “challenging behavior”[tw] OR “challenging behaviors”[tw])) | 2019-2024 |

*Note.* Asterisks (*) denote an integrated search, meaning all five databases were searched simultaneously.
